# Oxytocin Enhances the Neural Efficiency of Social Perception

**DOI:** 10.3389/fnhum.2019.00071

**Published:** 2019-03-11

**Authors:** Rachael Tillman, Ilanit Gordon, Adam Naples, Max Rolison, James F. Leckman, Ruth Feldman, Kevin A. Pelphrey, James C. McPartland

**Affiliations:** ^1^Department of Psychology, University of Maryland, College Park, College Park, MD, United States; ^2^Yale Child Study Center, School of Medicine, Yale University, New Haven, CT, United States; ^3^Department of Psychology, Bar-Ilan University, Ramat Gan, Israel; ^4^Department of Psychology, Interdisciplinary Center (IDC) Herzliya, Herzliya, Israel; ^5^Harrison-Wood Jefferson Scholars Foundation Professor, University of Virginia, Charlottesville, VA, United States

**Keywords:** ERP, EEG, face perception, oxytocin, emotion perception

## Abstract

Face perception is a highly conserved process that directs our attention from infancy and is supported by specialized neural circuitry. Oxytocin (OT) can increase accuracy and detection of emotional faces, but these effects are mediated by valence, individual differences, and context. We investigated the temporal dynamics of OT’s influence on the neural substrates of face perception using event related potentials (ERPs). In a double blind, placebo controlled within-subject design, 21 healthy male adults inhaled OT or placebo and underwent ERP imaging during two face processing tasks. Experiment 1 investigated effects of OT on neural correlates of fearful vs. neutral facial expressions, and Experiment 2 manipulated point-of-gaze to neutral faces. In Experiment 1, we found that OT reduced N170 latency to fearful faces. In Experiment 2, N170 latency was decreased when participant gaze was directed to the eyes of neutral faces; however, there were no OT-associated effects in response to different facial features. Findings suggest OT modulates early stages of social perception for socially complex information such as emotional faces relative to neutral. These results are consistent with models suggesting OT impacts the salience of socially informative cues during processing, which leads to downstream effects in behavior. Future work should examine how OT affects neural processes underlying basic components of social behavior (such as, face perception) while varying emotional expression of stimuli or comparing different characteristics of participants (e.g., gender, personality traits).

## Introduction

Oxytocin (OT) is a phylogenetically ancient neurohormone present in all mammals that exerts specific effects on social behavior (e.g., Gordon et al., 2011; Guastella and MacLeod, 2012). Face perception is an early developing, central facet of interpersonal interaction in humans subserved by specialized neural circuitry (Goren et al., 1975; Hershler and Hochstein, 2005). Many studies have provided compelling evidence that OT facilitates face and emotion recognition (see reviews by Van IJzendoorn and Bakermans-Kranenburg, 2012; Sharestani et al., 2013; Bethlehem et al., 2014). OT’s increased accuracy of facial expression recognition may partially reflect enhanced attention to the eyes (Gamer et al., 2010; Domes et al., 2012; Tollenaar et al., 2013); however, effects on accuracy and gaze are not evident in all studies (e.g., Guastella et al., 2009; Lischke et al., 2012b), and improved expression decoding can occur without changes in gaze behavior (Lischke et al., 2012a).

OT’s influence on face perception reflects action on specific brain networks involved in social perception, clarified by functional magnetic resonance imaging (fMRI) studies. Attenuated amygdala activity and connectivity in response to emotional faces has been the most common finding (e.g., Kirsch et al., 2005; Domes et al., 2010; Kanat et al., 2015). However, additional studies reveal a more complex account of the effects of OT on face perception and amygdala response, reflecting the influence of facial expression (Gamer et al., 2010), gender of participant (Domes et al., 2007, 2010), individual differences on measures of agreeableness and sociality (Groppe et al., 2013), and the sub-regions of the amygdala chosen for analysis (Gamer et al., 2010). OT has also been shown to affect activity in the fusiform gyrus (FG) and inferior frontal gyrus (IFG) during passive emotional face processing (Domes et al., 2007) and during evaluation of aversively-conditioned faces (Petrovic et al., 2008). Given a clear relationship to both amygdalar and FG activity and the substantial electrophysiology and neuroimaging literature dedicated to fear-related processes and faces (e.g., Adolphs, 2008), the current study focused specifically on the emotional expression of fear.

Several mechanisms have been put forward to explain the social effects of OT (Zink and Meyer-Lindenberg, 2012; Bethlehem et al., 2013; Gordon et al., 2013; Weisman and Feldman, 2013). One set of hypotheses argues that OT decreases anxiety *via* its modulation of amygdala-related neural circuits, yielding effects on social behavior secondary to reduced anxiety (Churchland and Winkielman, 2012). Others posit that OT shifts attention and approach biases toward positive social cues and thus improves emotion recognition and social behavior by modulating neural reward circuits (Strathearn et al., 2009; Groppe et al., 2013). The diverse effects of OT on social function have also been speculated to reflect modulation of neural connectivity, either within social brain circuitry (e.g., regions like the FG and superior temporal sulcus; Brothers, 1990) or across distributed brain systems (Bethlehem et al., 2013). A final set of hypotheses suggest that OT affects social behavior directly by increasing the salience of social information in the environment (Shamay-Tsoory et al., 2009; Bartz et al., 2011; Shamay-Tsoory and Abu-Akel, 2016) through influence on the amygdala and striatum, as well as the medial prefrontal cortex (MPFC) and social brain regions. The current study investigates mechanisms associated with these social salience hypotheses by examining the influence of OT on processing efficiency for social information.

At present, little is understood about the temporal dynamics of OT’s impact on face processing. For example, given the OT-associated changes in activation in both amygdala and FG, it is not known whether OT directly modifies early perceptual activity in the FG or increases activity *via* modulatory influences on other regions, such as the amygdala. Another gap in the understanding of OT’s effects on the brain involves processing speed, or efficiency, as indexed by latency of neural response. Social percepts, such as faces, have a relative advantage in terms of the efficiency with which they are processed relative to non-social percepts (Bentin et al., 1996; Hershler and Hochstein, 2005). Most studies have not applied methods with sufficient temporal resolution to investigate whether neural efficiency is modulated by OT.

Event-related brain potentials (ERPs) offer exquisite temporal resolution. This sensitivity enables specification of neural activity at temporally discrete stages of cognitive or perceptual processing, and it permits measurement of neural efficiency at each stage (i.e., the latency of an ERP component; McPartland et al., 2014). Face perception involves a rapidly-occurring cascade of distinct cognitive events marked by specific ERP components, advancing from low-level perception (e.g., attentional allocation; Hillyard and Anllo-Vento, 1998; Rossion and Caharel, 2011) to face structural encoding to higher-order perceptual processes, such as decoding affective state (Said et al., 2011). The P100 is a positive deflection over the occipital region that peaks between 80 and 120 milliseconds in response to any visual stimulus, indexing low-level, content-general visual perception in visual cortex (Regan, 1989). Studies have found increased P100 for emotional compared to neutral expressions, suggesting some facets of rapid emotional processing occur independently of later neural activity indexing face-selective processes (Eimer and Holmes, 2002; Holmes et al., 2003; Pourtois et al., 2005). Other studies have reported no such modulation (Mühlberger et al., 2009; Righi et al., 2012; Wynn et al., 2013), potentially reflecting variability among task demands and low-level image properties within stimulus sets (Rossion and Caharel, 2011; Rousselet and Pernet, 2011).

The N170 is a negative deflection over occipitotemporal scalp approximately 130–200 ms after perception of a visual event that marks face structural encoding (i.e., recognizing a face as such; Bentin et al., 1996). The N170 is differentially sensitive to individual facial features, with eyes and mouths eliciting greater amplitude than other facial features (Bentin et al., 1996; McPartland et al., 2010). With respect to N170 latency, intact faces evoke shortest latencies, followed by eyes then noses and mouths (Bentin et al., 1996). The N170 is modulated by visual attention to specific facial features, with fixations to the eye and mouth regions of the face eliciting enhanced N170 amplitudes and longer N170 latencies (McPartland et al., 2010). Several studies indicate that N170 response is modulated by emotional expression (e.g., Blau et al., 2007), though these effects may reflect alteration of face configuration rather than emotional information (Shibata et al., 2002). Neural generators of the N170 have been localized to occipitotemporal regions, including the FG (Itier and Taylor, 2004) and superior temporal sulcus (Wynn et al., 2013). An enhanced amplitude of the earlier posterior negativity (EPN), a negative wave peaking approximately 240–280 ms after presentation of a face over occipitotemporal regions, reflects facilitated perceptual processing of threatening faces compared with friendly or neutral faces (Sato et al., 2001; Schupp et al., 2004) and is thought to index attention to emotionally salient information. The EPN likely reflects cortical activity driven by amygdalar reciprocal feedback processes (e.g., Anderson and Phelps, 2001; Sabatinelli et al., 2005; Herbert et al., 2008). The N250 component and the EPN are similar; the N250 is measured between 200 and 300 ms over occipitotemporal regions or fronto-midline sites and reflects higher-order processing of complex facial information such as identity and affect (Sato et al., 2001; Wynn et al., 2013; Leleu et al., 2015).

To date, few studies have used ERPs to investigate the effects of OT on face perception. OT has been found to facilitate greater accuracy of familiarity judgments in a face recognition task as indexed by ERP components related to memory encoding and retrieval (FN400; Herzmann et al., 2013). This study did not indicate modulation of earlier perceptual components (P100, N170, P200). A second study, focusing on maternal attachment, revealed OT enhanced the vertex-positive-potential, a component likely representing dipolar activity consistent with the N170, for happy and disgusted faces among individuals with more adaptive attachment recollections (Huffmeijer et al., 2013). Recent work has investigated OT’s influence on the neural dynamics of adult’s processing of infant faces, varying the parenting status of the participants (Waller et al., 2015; Rutherford et al., 2017; Peltola et al., 2018) and familiarity of the infant face stimuli (Waller et al., 2015). In fathers who viewed faces of unfamiliar children, familiar children, and their own children, OT attenuated later-stage ERP components, reflective of perceptual memory representations and emotional valence (Waller et al., 2015). Among mothers of 1-year-old infants, OT enhanced the N170 for faces of emotional infants and adults (Peltola et al., 2018), while among non-parent women, OT only enhanced the later P300 component (associated with attention allocation to stimuli; Ritter and Ruchkin, 1992; Luck, 2005), for only infant faces relative to adult faces (Rutherford et al., 2017).

These studies demonstrate the utility of ERPs for measuring the effects of OT on face perception; however, they underscore the complexity of OT’s effects and raise several important questions. Two studies embedded face processing in the context of complex cognitive tasks involving working memory and executive function; as cognitive load is recognized to influence social perception (e.g., Puce et al., 1999), it is not clear to what extent these results are reflective of face processing in more standard, passive viewing contexts. Second, prior work has focused on comparisons of attachment-related stimuli (e.g., infants vs. adult faces). While these studies are important for understanding underlying mechanisms of OT’s influence on parent-child bond, they do not directly assess OT’s impact on face perception more broadly. Third, although the interaction of point of gaze to the face and the effects of OT is well recognized (Tollenaar et al., 2013), prior ERP studies of face perception did not investigate the influence of gaze. In this way, it is unclear whether variations in gaze associated with performance of cognitive tasks (e.g., focusing on a particular aspect of faces to facilitate task performance) may have affected results. Furthermore, direct investigation of OT modulated brain response to specific facial features (e.g., the eyes or mouth instead of holistic face perception) has only been examined with fMRI, revealing OT-associated reductions in amygdala activation when attending to the eye region of angry faces and to the mouth region of happy faces (Kanat et al., 2015). These results emphasize the need for additional work investigating neural activation to a variety of facial features (e.g., eyes and mouth) depicting a variety of expressions (e.g., neutral, fearful, happy) using different methodology (i.e., ERP).

The current study is designed to investigate the effects of OT on face processing in conventional face perception paradigms (e.g., Bentin et al., 1996) without significant cognitive load. Specifically, we sought to assess the influence of OT at different stages of early face processing (the P100, N170, and the EPN) and to determine whether these effects were impacted by subtle changes in social information such as expression and attention to distinct facial features. This is the first examination of the influence of OT on the early stages of face processing in a passive viewing paradigm (without an explicit task or comparison of infant faces) and the first to examine the interaction between OT administration and point of gaze on face-related ERP activity.

In a randomized, double-blind, and placebo-controlled design, brain response in typical adults was recorded during two experiments examining socially important aspects of face perception: (a) emotional face perception (fear and neutral); and (b) point of gaze to the face (eyes, nose, and mouth). We hypothesized that OT would enhance early indices of face perception, manifest in increased amplitude or reduced latency in the aforementioned components. As suggested by the social salience hypothesis (Shamay-Tsoory and Abu-Akel, 2016), we predicted that OT would exert a greater influence on socially salient information, i.e., fearful expressions and attentional focus on the eyes, compared to less socially salient information (i.e., neutral expressions and attentional focus on the nose or mouth). In exploratory analyses, we sought to investigate the relationships between behavioral characteristics and OT-associated neural response to faces. Thus, behavioral measures of social function and trait anxiety were correlated with ERP components indexing effects of OT.

## Materials and Methods

### Participants

Twenty-one typically developing adult males aged 19–32 years (mean age = 25.2, SD = 3.68) were recruited from the New Haven community. Exclusionary criteria included seizures, neurological disease, history of head injury, sensory motor impairment, active psychiatric disorder, learning/language disability, family history of autism spectrum disorder (ASD), and anti-convulsant medications. Four participants were left-handed and all had normal or corrected-to-normal vision. This study was carried out in accordance with the recommendations of Yale University Institutional Review Board with written informed consent from all subjects. All subjects gave written informed consent in accordance with the Declaration of Helsinki. The protocol was approved by the Yale University Institutional Review Board.

### Procedure and Stimuli

Participants completed two EEG sessions on separate days with an interval of at least 3 days and no more than 2 weeks in a double-blind, placebo-controlled within-subject design. Forty-five minutes prior to the beginning of each EEG session, participants received a nasal spray that contained either active OT, 60 international units per milliliter (IU/ml), or a placebo. The OT nasal spray was prepared by the research pharmacy at Yale New Haven Hospital using OT, USP (Medisca, Las Vegas, NV, USA). Placebo and OT spray containers were prepared to look identical, and researchers and participants were blind to the content of the spray. Participants received a dose of 24 IU (four puffs overall, two per each nostril, one puff administered a 6 IUs dose), in accordance with most studies of intranasal OT in adults (e.g., Domes et al., 2007; Guastella et al., 2009). The administration order of OT and placebo were randomly counterbalanced by the pharmacy across participants. Consistent with the gold-standard for human OT studies, the EEG tasks took place between 45 and 95 minutes post administration. Several studies have shown that the effects of intranasal OT administration as reflected in elevations in salivary OT levels begin as soon as 15 minutes following administration and do not diminish even 4 hours following (Huffmeijer et al., 2013; Weisman and Feldman, 2013). However, this timing is dependent on loci of concentrations measured, as OT increase and latencies are different for OT concentrations in plasma and cerebral spinal fluid (Striepens et al., 2013). Prior to the first EEG session and immediately following OT administration, two self-report measures were administered to assess aspects of participants’ social functioning and level of anxiety (State-Trait Anxiety Inventory; STAI; Spielberger et al., 1983; the Adult Self-Report form of the Social Responsiveness Scale; SRS; Constantino, 2002; Constantino and Todd, 2003, respectively).

In this within-subject design, EEG data were collected from two face processing paradigms, which were run consecutively one after another during every visit. Experiment 1 was presented an average of 50.3 minutes (*SD* = 3.59) after nasal inhalation, and Experiment 2 was presented an average of 85.1 minutes (*SD* = 5.95) after nasal inhalation. An additional experiment unrelated to face processing was conducted in between the current study’s Experiment 1 paradigm and Experiment 2 paradigm. Findings from this unrelated experiment are not presented in the current manuscript. In Experiment 1, participants viewed 70 distinct computer-generated, dynamic, grayscale faces (35 female faces and 35 male faces; Naples et al., 2014). Experiment 1 lasted for 15 minutes with 146 total trials in random sequence (70 neutral to fearful faces, 70 fearful to neutral faces, and three targets). Each trial started with a central fixation crosshair presented for a jittered duration (between 200 and 300 ms); a centrally presented static face followed displaying either a neutral or fearful expression for 500 ms. At the 500 ms time point, the face changed expression to either fearful or neutral in a naturalistic, animated, and veridical movement. The face stimuli were presented for a total of 1,000 ms; ERPs were only segmented to the initial, static face (displayed for 500 ms) for both neutral and fearful conditions. Participants pressed a button upon detection of randomly interspersed target stimuli (white balls) to ensure attention. We analyzed the static portion of the trials based to enable extraction of the specific ERP components of interest related to face and emotion perception. The face stimuli following the target stimuli, a total of three trials, were excluded from analysis.

In Experiment 2, participants were presented 157 distinct, grayscale digital images of neutral faces (80 male, 77 female; from the Center for Vital Longevity Face Database; Minear and Park, 2004) and 40 houses. Experiment 2 lasted for 7 minutes with 197 total trials in random sequence (157 faces and 40 houses). In each trial: a fixation crosshair was presented for jittered duration (between 100 and 500 ms) and was followed immediately by a randomly selected face or house stimulus for 500 ms. A blank screen was then presented after the stimulus for 700 ms. Visual attention was manipulated by changing the vertical position of the fixation crosshair to either the (a) upper, (b) central, or (c) lower regions of the stimulus. The horizontal position of the crosshair was held constant, and vertical position was equiprobable and varied randomly among trials. In this way, participant gaze was directed to the eyes, nose, or mouth of the onscreen face. Participants pressed a button upon detection of randomly interspersed target stimuli (seven faces, four houses, nine crosshairs; shaded red) to ensure attention. Attention tasks of this nature have been effective in manipulating point of gaze and demonstrating consequent modulation of behavior and brain response in previous face processing research (Gamer et al., 2010; McPartland et al., 2010). Target trials were excluded from analysis. House stimuli were included to enable us to validate the face sensitivity N170, i.e., enhanced amplitude to faces. Because we did not have hypotheses about the influence of OT on these non-social stimuli, we excluded them from analyses of OT effects to maximize statistical power for pre-specified comparisons of interest.

All stimuli were presented on a uniform gray background on an 18-inch color monitor (60 Hz, 1,024 × 768 resolution) with E-Prime 2.0 software (Schneider et al., 2002) at a viewing distance of 75 cm in a sound attenuated room with low ambient illumination. All faces were cropped within an oval frame to remove non-face features (Gronenschild et al., 2009). EEG was recorded continuously at 500 Hz using NetStation 4.2.1. A 128-electrode Hydrocel Geodesic Sensor Net (Electrical Geodesics, Inc., Eugene, OR; Tucker, 1993) was fitted on the participant’s head according to manufacturer’s specifications. Impedances were kept below 40 kΩ.

### EEG Data Recording and Analysis

All data were low-pass filtered offline at 30 Hz prior to segmentation. For Experiment 1, filtered data were segmented to an epoch lasting from 100 ms before to 500 ms after stimulus onset, and for Experiment 2, filtered data were segmented to an epoch lasting from 100 ms before to 900 ms after stimulus onset. For both experiments, artifact detection settings were set to 200 μV for bad channels, 140 μV for eye blinks, and 100 μV for eye movements. Channels with artifacts on more than 15 or 40% of trials were marked as bad channels and replaced through spline interpolation. Segments that contained eye blinks, eye movement, or more than 10 bad channels were marked as bad and excluded. Participants with more than 15% of bad channels were excluded from analysis. All data were re-referenced to an average reference and baseline corrected to the 100 ms pre-stimulus epoch. Trial-by-trial data were subsequently averaged at each electrode for each condition, e.g., “fear” and “neutral,” separately for every individual. After EEG processing, the final sample included 21 participants for Experiment 1 and 19 participants for Experiment 2.

**Figure 1:**
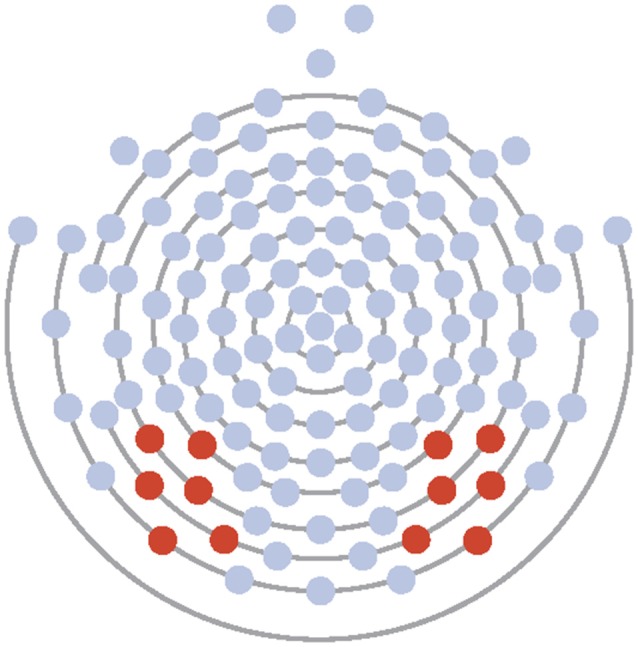
Electrode layout for Experiments 1 and 2 (electrodes 58, 59, 64, 65, 68, 69 on the left and 89, 90, 91, 94, 95, 96 on the right).

Electrodes of interest were selected based on maximal observed amplitude of the N170 to faces and to conform to those used in previous research (McPartland et al., 2004). Data from Experiment 1 and 2 were averaged across six electrodes over the left (58, 59, 64, 65, 68, 69) and right (89, 90, 91, 94, 95, 96) lateral posterior scalp (see Figure 1). Time windows for ERP analysis were first chosen by visual inspection of grand averaged data. Next, time windows were modified by manual investigation of each individual’s average and generated to ensure capture of only the peak of interest. For all participants, resultant time windows were 72–110 ms for the P100, from 110 to 210 ms for the N170, and from 200 to 300 ms for the EPN in Experiment 1. In Experiment 2, resultant time windows were 72–146 ms for the P100, from 110 to 250 ms for the N170, and from 200 to 300 ms for the EPN in Experiment 2. Maximum peak amplitude and latency to the maximal peak were calculated for the P100 within the time window for Experiments 1 and 2. Minimum peak amplitude and latency to the minimal peak were calculated for the N170 within its respective time window for Experiment 1. Due to the morphology and lack of clear peak of the EPN in Experiment 1, mean amplitude for the component was calculated. For Experiment 2, minimum peak amplitude and latency to the minimal peak were calculated for both the N170 and EPN within their respective time windows. Component information was exported to SPSS for analysis.

For Experiment 1, the following dependent variables were analyzed using a three-factor repeated measures ANOVA: treatment (OT, placebo); condition (fear, neutral); and hemisphere (left, right). For Experiment 2, the following dependent variables were analyzed using a three-factor repeated measures ANOVA: treatment (OT, placebo); condition (face with participant gaze directed to the eyes, nose, or mouth); and electrode hemisphere (left, right). The Greenhouse-Geiser correction for sphericity was applied. *Post hoc* analyses were computed using simple effects analyses and Bonferroni-corrected for multiple comparisons. Bivariate logistic regressions were conducted for specific latency difference scores to explore significant treatment and condition interactions.

## Results

### Experiment 1

#### P100 Amplitude

There were no significant main effects observed for treatment, condition, or hemisphere (all *F*s ≤ 2.20, all *p*s > 0.15) and no significant two- or three-way interactions (all *F*s ≤ 2.08, all *p*s > 0.17).

#### P100 Latency

There were no significant main effects observed for treatment, condition, or hemisphere (all *F*s ≤ 2.86, all *p*s > 0.11 and no significant two- or three-way interactions (all *F*s ≤ 1.79, all *p*s ≥ 0.20).

#### N170 Amplitude

**Figure 2:**
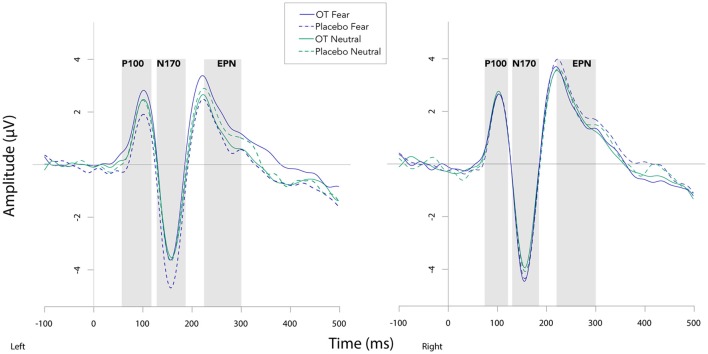
Grand averaged waveforms in the left and right hemisphere (left and right panels, respectively) elicited by fearful and neutral faces for participants after oxytocin and for participants after placebo. OT, oxytocin; ms, milliseconds; μV, microvolts.

**Figure 3:**
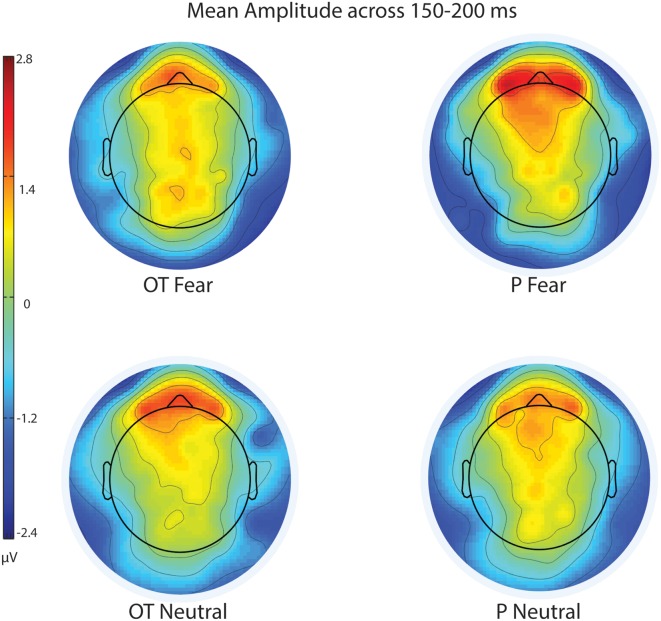
Scalp topography of mean voltage across the N170 time window (150–200 ms) for treatment and facial expression conditions for Experiment 1. OT, oxytocin; P, placebo; ms, milliseconds; μV, microvolts.

Fearful faces elicited an enhanced N170 amplitude relative to neutral faces (see Figures 2, 3; *F*_(1,20)_ = 6.54, *p* = 0.02, $\eta_{\text{p}}^{2}$ = 0.25). There were no other significant main effects observed for treatment (*F*_(1,20)_ = 0.30, *p* = 0.59, $\eta_{\text{p}}^{2}$ = 0.02) and hemisphere (*F*_(1,20)_ = 1.58, *p* = 0.22, $\eta_{\text{p}}^{2}$ = 0.07). Additionally, none of the remaining two- or three-way interactions were significant (all *F*s ≤ 3.95, all *p*s ≥ 0.06).

#### N170 Latency

**Figure 4:**
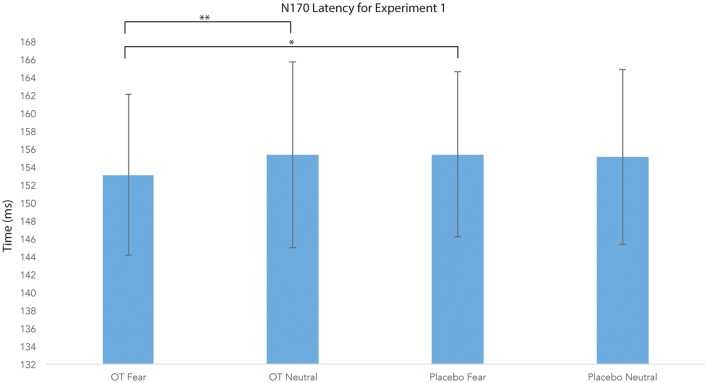
N170 latency as a function of emotion and treatment (hemisphere collapsed) for Experiment 1. A single asterisk indicates the statistically significant relationship (uncorrected for multiple comparisons) and double asterisks indicate statistically significant relationship at the Bonferroni-corrected level. Error bars indicate standard deviation. OT, oxytocin; ms, milliseconds.

For N170 latency (see Figures 2, 4 and Table 1), a treatment by condition interaction (*F*_(1,20)_ = 4.85, *p* = 0.04, $\eta_{\text{p}}^{2}$ = 0.20) was observed. In order to explore this interaction, we compared simple effects of treatment for condition, which revealed that when subjects viewed fearful faces, N170 latency was shorter when subject had inhaled OT compared to when subjects had inhaled the placebo (mean difference i − j = −1.93, *p* = 0.04). This relationship did not survive correction for multiple comparisons (Bonferroni-adjusted level = 0.0125). Comparison of simple effects of treatment for condition revealed that when subjects inhaled OT, N170 latency to fearful faces was significantly shorter compared to neutral faces (mean difference i − j = −2.50, *p* = 0.004), whereas no such difference was observed when subject inhaled placebo (mean difference i − j = −0.095, *p* = 0.92). This relationship survived correction for multiple comparisons (Bonferroni-adjusted level = 0.0125).

**Table 1:** Average means and standard deviations for N170 latency for Experiment 1.

	Mean (ms)	Standard deviation (ms)
Oxytocin fear left	154.16	9.93
Oxytocin fear right	154.06	10.60
Oxytocin neutral left	157.54	10.88
Oxytocin neutral right	155.68	13.88
Placebo fear left	156.51	9.51
Placebo fear right	155.57	10.83
Placebo neutral left	156.27	10.51
Placebo neutral right	155.62	11.57

There were no significant main effects observed for treatment and condition in the two- and three-way model and hemisphere in the three-way model (all *F*s ≤ 4.23, all *p*s ≥ 0.05). Additionally, we observed no other significant interactions for remaining two- or three-way analyses (all *F*s ≤ 1.46, all *p*s ≥ 0.24).

#### EPN Amplitude

**Figure 5:**
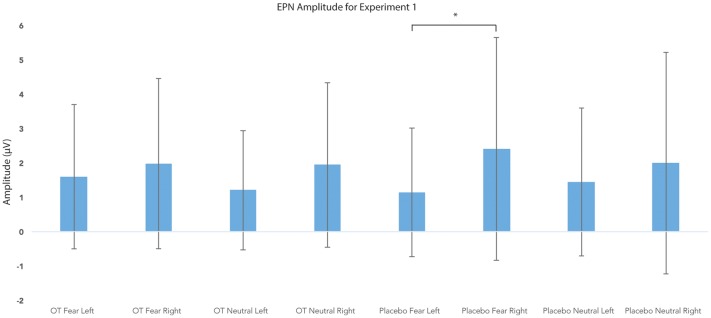
Earlier posterior negativity (EPN) amplitude as a function of emotion and treatment in the left and right hemispheres for Experiment 1. The asterisk indicates the statistically significant relationship (uncorrected for multiple comparisons). Error bars indicate standard deviation. OT, oxytocin; μV, microvolts.

For EPN amplitude (see Figures 2, 3, 5 and Table 2), there was a treatment by condition by hemisphere interaction (*F*_(1,20)_ = 6.37, *p* = 0.02, $\eta_{\text{p}}^{2}$ = 0.24). Comparison of simple effects of treatment revealed that when subjects had placebo and viewed fearful faces, the mean EPN amplitude was enhanced (more negative) in the left hemisphere than the right hemisphere (mean difference i − j = −1.26, *p* = 0.029). Visual inspection of the pattern showed that the means of EPN amplitude for neutral faces did not exhibit lateralization across OT and placebo. However, fearful faces elicited left lateralized EPN amplitude when individuals inhaled placebo; whereas when they inhaled OT, they exhibited no significant lateralization difference in EPN amplitude. This relationship did not survive correction for multiple comparisons (Bonferroni-adjusted level = 0.0125).

**Table 2:** Average means and standard deviations for earlier posterior negativity (EPN) mean amplitude for Experiment 1.

	Mean (μV)	Standard deviation (μV)
Oxytocin fear left	1.59	2.14
Oxytocin fear right	1.97	2.53
Oxytocin neutral left	1.21	1.78
Oxytocin neutral right	1.94	2.45
Placebo fear left	1.14	1.91
Placebo fear right	2.40	3.32
Placebo neutral left	1.44	2.20
Placebo neutral right	2.00	3.31

There were no significant main effects observed for treatment, condition, and hemisphere (all *F*s ≤ 3.09, all *p*s ≥ 0.09). Additionally, no other significant interactions were observed (all *F*s ≤ 1.46, all *p*s ≥ 0.24).

### Experiment 2

#### P100 Amplitude

There was a main effect of hemisphere (*F*_(1,18)_ = 9.34, *p* = 0.007, $\eta_{\text{p}}^{2}$ = 0.34). Comparison of simple effects of hemisphere revealed that the right hemisphere elicited enhanced amplitudes relative to the left (mean difference i − j = −0.91, *p* = 0.007). There were no significant main effects observed for treatment or condition and no significant interactions observed between treatment, condition, and hemisphere (all *F*s ≤ 2.73, all *p*s ≥ 0.12).

#### P100 Latency

There were no significant main effects observed for treatment, condition, or hemisphere (all *F*s ≤ 3.62, all *p*s ≥ 0.05). Additionally, no significant interactions were observed (all *F*s ≤ 2.86, all *p*s ≥ 0.07).

#### N170 Amplitude

There were no significant main effects observed for treatment, condition, or hemisphere (all *F*s ≤ 2.06, all *p*s ≥ 0.15). Additionally, we observed no significant interactions for two-way or three-way interactions (all *F*s ≤ 0.84, all *p*s ≥ 0.37). Paired sample *t-tests* revealed that face stimuli elicited a significantly larger amplitude relative to house stimuli in the right and left hemispheres (*t*_(18)_ = −6.23, *p* ≤ 0.001 and *t*_(18)_ = −8.66, *p* ≤ 0.001, respectively).

#### N170 Latency

**Figure 6:**
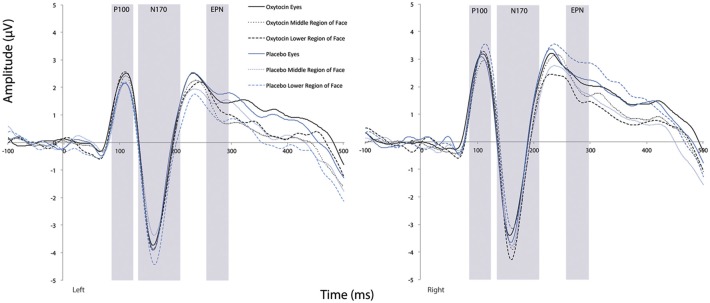
Grand averaged waveforms in the left and right hemisphere (left and right panels, respectively) elicited by the eye region, middle region, and lower face regions of faces for participants after oxytocin and for participants after placebo. ms, milliseconds; μV, microvolts.

There was a main effect of condition (*F*_(1.41,25.43)_ = 4.14, *p* = 0.04, $\eta_{\text{p}}^{2}$ = 0.19). Comparison of simple effects of condition revealed that the eye region of the face elicited shorter N170 latency compared to the mouth region of the face (mean difference i − j = −2.67, *p* = 0.008). There were no significant main effects observed for treatment or hemisphere (all *F*s ≤ 0.27, all *p*s ≥ 0.61). Additionally, none of the two- or three-way interactions were significant (all *F*s ≤ 1.09, all *F*s ≥ 0.34; see Figure 6).

#### EPN Amplitude

There was a main effect of hemisphere (*F*_(1,18)_ = 4.78, *p* = 0.04, $\eta_{\text{p}}^{2}$ = 0.21). Comparison of simple effects of hemisphere revealed that the right hemisphere elicited enhanced amplitude relative to the left (mean difference i − j = −0.86, *p* = 0.04). There were no significant main effects observed for treatment or condition and no significant interactions observed between treatment, condition, and hemisphere (all *F*s ≤ 1.74, all *p*s ≥ 0.19).

#### EPN Latency

There were no significant main effects observed for treatment, condition, or hemisphere (all *F*s ≤ 2.26, all *p*s ≥ 0.15). Additionally, we observed no significant interactions for two-way or three-way interactions (all *F*s ≤ 2.18, all *p*s ≥ 0.14).

### Behavioral Correlations

**Figure 7:**
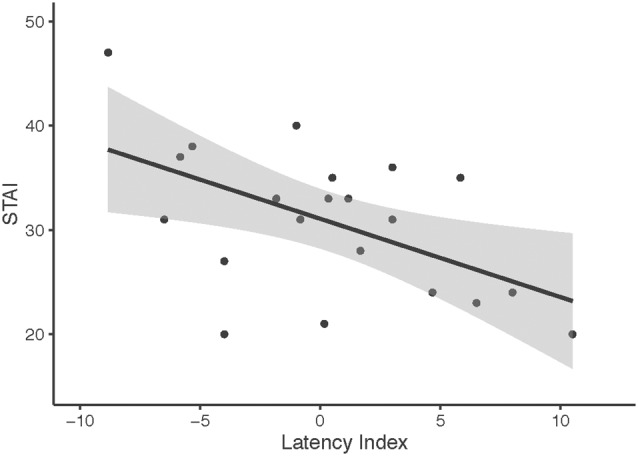
OT-associated change in N170 latency to neutral faces across hemisphere and self-reported trait anxiety scores (STAIs). Higher STAI scores represent higher trait anxiety. Latency index was calculated by creating a difference score between the N170 latencies to neutral faces collapsed across hemisphere (N170 latency after OT administration minus Nl70 latency after placebo administration).

In order to investigate the significant interaction between condition and treatment in Experiment 1, we calculated OT-associated change in N170 latency (collapsed across hemispheres). For fearful faces, there was no relationship between OT-associated change in N170 latency and the SRS (*r* = −0.002, *p* = 0.99) nor the STAI (*r* = 0.08, *p* = 0.74). For neutral faces, OT-associated change in N170 latency was negatively correlated with the STAI (*r* = −0.53, *p* = 0.013); shorter latencies in the OT condition were associated with higher levels of trait anxiety, while longer latencies in the OT condition were observed for lower levels of trait anxiety (see Figure 7). This relationship did not survive correction for multiple comparisons (Bonferroni-adjusted level = 0.0125).

There was no significant relationship between the OT-associated change in N170 latency for neutral faces and the SRS (*r* = −0.28, *p* = 0.23). There was one outlier among the SRS scores. This outlier was within acceptable limits for participant self-reports of social responsiveness; nevertheless, to understand its influence on our results, we removed the outlier and reanalyzed our findings. With the SRS outlier score removed, a comparable pattern of results was observed for fearful (*r* = 0.07, *p* = 0.79) and neutral faces (*r* = −0.14, *p* = 0.56).

## Discussion

This study investigated the temporal dynamics of OT’s influence on the neural substrates of face perception using ERPs. Experiment 1 investigated its relationship with fearful expression, and Experiment 2 focused on point of gaze to the face. In Experiment 1, administration of OT elicited a shorter N170 latency to fearful faces, indicating increased processing efficiency for negative emotional information at early perceptual stages. EPN amplitude to fearful faces was less lateralized during administration of OT.

In Experiment 2, the P100 and EPN amplitudes were enhanced overall in the right hemisphere. Consistent with prior research (Bentin et al., 1996), the eye region of the face elicited shorter N170 latency compared to the mouth region of the face. However, no effects of OT administration were observed. Across experiments, no effects of OT administration were observed for early occurring, exogenous sensory components (i.e., P100).

The temporal resolution of EEG provides key information regarding the stages of influence of OT. OT did not affect low-level visual processing at the P100. In Experiment 1, effects were evident at subsequent ERP components (N170, EPN) associated with social- affective perception. Counter to theories suggesting that OT may enhance domain-general sensory vigilance (Harari-Dahan and Bernstein, 2014), these results suggest OT’s effects are most pronounced for social-affective brain functions.

At the N170, OT administration was associated with shorter latency to fearful, but not neutral, faces. We interpret this reduced lag as evidence for enhanced neural efficiency for emotional information processing under OT. Studies have explored the functional significance of the N170 and have demonstrated that the component’s latency predicts accuracy and speed of face recognition (Herzmann et al., 2010; Rousselet et al., 2014). Thus, we conceptualize OT’s role in face processing as facilitating creation of the structural representation of a face, which in turn could lead to more rapid social cognition. Our findings are consistent with prior studies showing that OT enhances the impact of aversive social stimuli (Striepens et al., 2012), increases neural activation to both positive and negative social cues (Groppe et al., 2013), and modulates early, face-sensitive ERPs in response to social emotional signals (Peltola et al., 2018).

In Experiment 1, the amplitude of the EPN component varied as a function of OT, facial expression, and hemisphere, such that when individuals inhaled placebo, fearful faces elicited increased lateralization in the left hemisphere. This finding does not align with previous work demonstrating selectively enhanced EPN amplitude to emotionally salient relative to neutral faces, typically in the right hemisphere. Work focusing on the brain lateralization of holistic vs. part-based processing of emotional facial expressions has suggested that N170 and EPN components are modulated by expression of whole faces, but not by separate regions of faces, in the right hemisphere (Calvo and Beltrán, 2014). Given that facial expression recognition requires holistic and analytic mechanisms (Tanaka et al., 2012), OT’s reduction in lateralization could reflect a shift or balance in typical expression encoding and emotional assessment processes; however, more research focused on the effects of OT on face processing and ERP lateralization is needed to support this interpretation.

Distinct effects at the N170 (shorter latency) and the EPN (attenuated lateralization) reflect the complex modulatory role OT plays in social processing. One potential explanation of these results is that OT first enables us to quickly assess a potentially threating situation or a situation that relays emotional content, plausible considering its evolutionary-conservation (Gordon et al., 2011). Once the risk has been assessed as low, OT can then modulate greater motivation or approach orientation. This explanation helps disentangle the variable and context- dependent nature of OT’s effects (Bartz et al., 2011; Leppanen et al., 2017). Importantly, none of the components exhibited enhanced amplitudes or shorter latencies for fearful relative to neutral faces in the placebo condition. These results are not in line with our hypotheses and reflect the complexity of the neural underpinnings of emotional face perception. As discussed by Vuilleumier and Pourtois (2007), there have been mixed findings on modulation of the N170 by emotional expression. Despite multiple studies indicating that the N170 is modulated (in a non-selective manner) by the emotional valence of a face (Campanella et al., 2002; Pizzagalli et al., 2002; Batty and Taylor, 2003; Eger et al., 2003; Ashley et al., 2004; Miyoshi et al., 2004), it is most commonly thought to represent an early perceptual stage reflective of focus and attention rather than emotion perception (Carmel and Bentin, 2002; Jemel et al., 2003). Modulation of EPN amplitude by threatening faces, as compared with friendly or neutral faces, has also been reported (Sato et al., 2001; Schupp et al., 2004), but we did not observe these differences in the current study regardless of OT administration.

OT did not modulate neural response when participant’s attention was directed toward different regions of neutral faces. Prior behavioral research demonstrated that OT increases attention to the eyes during passive viewing of neutral and emotional facial stimuli (Guastella et al., 2008; Harari-Dahan and Bernstein, 2014; Andari et al., 2010). In an fMRI analog to Experiment 2, researchers manipulated point of gaze by presenting neutral, happy, and fearful faces shifted upward or downward relative to fixation (Gamer et al., 2010). They found that OT increased proportion of fixation changes toward eye regions across all expressions, and this activity was related to enhanced amygdala activity and increased functional connectivity between the amygdala and the superior colliculi. An fMRI study (Kanat et al., 2015), constrained fixation and focused solely on initial neural reactivity to subliminal emotional (happy and angry) facial features (eyes and mouth). OT reduced amygdala responses when attending to salient emotional facial features. Our study also targeted neural responses to initial fixation on facial features; however, these facial features did not have affective content.

In an exploratory analysis, we found suggestive evidence that OT-associated change in N170 latency was modulated by trait anxiety. Individuals with higher levels of anxiety showed increased responsivity to OT in terms of N170 latency to neutral faces. Evident to neutral but not fearful faces, the association may suggest the differential influence of OT in more anxious individuals when perceiving neutral or ambiguous social information. This finding is consistent with results that have suggested the effects of OT are more evident on challenging tasks (e.g., inferring mental state from nuanced facial expressions, Domes et al., 2007, 2013) and among individuals with anxiety and emotion regulation difficulties (Labuschagne et al., 2010, 2012; Quirin et al., 2011). Due to the exploratory nature of this relationship, more research is required to confirm this pattern.

Several limitations of this research raise key questions for future investigations. Since the mechanism by which OT is delivered to the brain following intranasal administration has not yet been fully established (Leng and Ludwig, 2016), researchers have called for caution when interpreting OT’s effects following nasal administration (Miller, 2013). Despite these concerns, there is evidence that intranasal OT administration can indeed lead to cerebral spinal fluid OT elevations (e.g., Striepens et al., 2013; Beard et al., 2018) and exude significant central effects (Gordon et al., 2013; Quintana and Woolley, 2016). Additionally, the different interval between OT administration and the onset of Experiment 1 and Experiment 2 is a possible limitation to the current results. As the effects of intranasal OT are considered to peak 45–70 minutes following administration (Spengler et al., 2017) and may plateau until 2 hours following administration (Weisman et al., 2012), we expect this limitation to have a small impact on our results.

The fearful and neutral faces used here do not permit extrication of the influence of general emotionality from negative valence. We chose to study fear because of its well- understood effects in neuroscience and ERP research. It will be helpful in future research to examine other emotions to clarify whether the effects observed here reflect general influence on emotion perception or a fear-specific effect. Additionally, given evidence of OT-mediated attractiveness biases (for a review, see Hurlemann et al., 2017), determining how attractiveness of faces may influence OT’s effects on the neural substrates of face processing is an important next step to understanding OT’s effects. In Experiment 2, we manipulated visual attention using crosshairs but did not directly measure gaze to the face. Given recent technological advances integrating EEG with eye-tracking, direct confirmation of point of gaze or ERPs elicited during passive viewing (EFRPs) can provide more reliable information about the relationship between gaze, face perception, and OT.

As this study was conducted in a relatively, small male only sample, the specificity of our results to the entire population are unknown. Future studies will need to replicate the presented findings and extend the demographics of the current sample. Future work could also be designed to answer questions regarding dose effects of a single administration of OT, and considering the context-dependent manner by which OT acts (Bartz et al., 2011), to investigate OT’s effects on different types of faces (e.g., faces from in- or out-groups members; different sexes of faces, faces of family members De Dreu et al., 2011; Herzmann et al., 2013). Another limitation of our methods is that ERP components may reflect enduring deflections (relative to a neutral baseline) from preceding components; in this way, differences evident at the EPN may be partially reflective of activity originating from processes indexed by the N170.

In summation, we demonstrate here, that OT modulates neural response at the earliest stages of social perception but not in prior, domain-general sensory processes. Although OT was associated with enhanced processing of affective stimuli at stages typically associated with structural encoding in the FG and superior temporal sulcus, OT also affected neural response to fearful faces at a subsequent component associated with emotion decoding reflective of downstream modulation by the amygdala. However, our findings indicate that the impact of OT on ERP indices of face perception is limited as only two of our comparisons revealed OT modulated effects. Taken together, these results provide support for theoretical accounts proposing that OT modulates social behavior by enhancing social salience mechanisms (Bethlehem et al., 2014; Leppanen et al., 2017). This model suggests that diverse and contextually dependent effects on social behavior reflect OT’s influence on dopaminergic and serotonergic brain circuitry across multiple functional domains, spanning anxiety, reward sensitivity, and attribution of social salience (Churchland and Winkielman, 2012; Dölen et al., 2013; Skuse and Gallagher, 2009; Ellenbogen, 2018). In ongoing work, it will be crucial to apply multimodal methods (e.g., eye-tracking, EEG) and include a variety of social and non-social stimuli of differing affective content within the same paradigm to elucidate OT’s complex modulatory role in information processing and its therapeutic potential.

## Data Availability

The datasets generated for this study are available on request to the corresponding author.

## Author Contributions

All authors contributed to the design of the experiments. RT, IG, JL, and MR collected the data. RT, IG, MR, AN, and JM analyzed the data. All authors contributed to writing the manuscript.

## Conflict of Interest Statement

The authors declare that the research was conducted in the absence of any commercial or financial relationships that could be construed as a potential conflict of interest.
